# 5-(3-Nitro­benz­yl)-1,3,4-thia­diazol-2-amine

**DOI:** 10.1107/S1600536809049654

**Published:** 2009-11-25

**Authors:** Samir A. Carvalho, Larisse O. de Feitosa, Edson F. da Silva, Edward R. T. Tiekink, James L. Wardell, Solange M. S. V. Wardell

**Affiliations:** a Fundação Oswaldo Cruz, Instituto de Tecnologia em Fármacos - Far-Manguinhos, Laboratório de Síntese IV, 21041-250 Rio de Janeiro, RJ, Brazil; b Instituto de Química, Universidade Federal do Rio de Janeiro, 21949-900 Rio de Janeiro, RJ, Brazil; c Department of Chemistry, University of Malaya, 50603 Kuala Lumpur, Malaysia; d Centro de Desenvolvimento Tecnológico em Saúde (CDTS), Fundação Oswaldo Cruz (FIOCRUZ), Casa Amarela, Campus de Manguinhos, Av. Brasil 4365, 21040-900, Rio de Janeiro, RJ, Brazil; e CHEMSOL, 1 Harcourt Road, Aberdeen AB15 5NY, Scotland

## Abstract

In the title mol­ecule, C_9_H_8_N_4_O_2_S, the dihedral angle between the thia­diazole and benzene rings is 73.92 (8)° and the thia­diazole group S atom is orientated towards the benzene ring, the central S—C—C—C torsion angle being 45.44 (18)°. In the crystal, supra­molecular tapes mediated by N—H⋯N hydrogen bonds and comprising alternating eight-membered {⋯HNCN}_2_ and 10-membered {⋯HNH⋯NN}_2_ synthons are formed along [010]. The tapes are consolidated into a three-dimensional network by a combination of C—H⋯O, C—H⋯S and C—H⋯π inter­actions

## Related literature

For background to the biological inter­est of 1,3,4-thia­diazo­les, see: Thomasco *et al.* (2003 ▶); Oruç *et al.* (2004 ▶); Moise *et al.* (2009 ▶); Amir *et al.* (2009 ▶). For the development of anti-trypanosomal compounds, see: Carvalho *et al.* (2004 ▶); Boechat *et al.* (2006 ▶); Boechat *et al.* (2008 ▶); Carvalho *et al.* (2008 ▶); Poorrajab *et al.* (2009 ▶) Riente *et al.* (2009 ▶). For the synthesis, see: Turner *et al.* (1988 ▶).
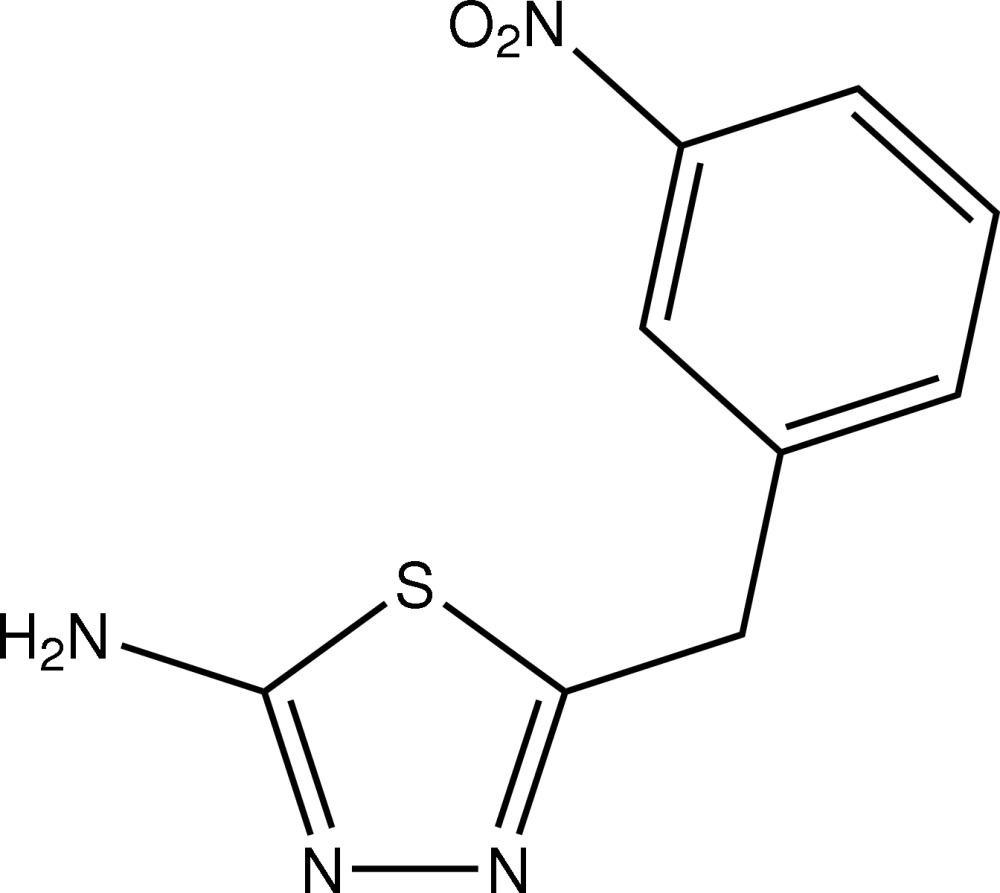


## Experimental

### 

#### Crystal data

C_9_H_8_N_4_O_2_S
*M* *_r_* = 236.26
Triclinic, 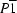

*a* = 5.0878 (2) Å
*b* = 5.6213 (3) Å
*c* = 17.8035 (9) Å
α = 80.980 (3)°
β = 85.677 (3)°
γ = 79.855 (3)°
*V* = 494.42 (4) Å^3^
*Z* = 2
Mo *K*α radiation
μ = 0.32 mm^−1^
*T* = 120 K
0.38 × 0.20 × 0.09 mm

#### Data collection

Nonius KappaCCD area-detector diffractometer
Absorption correction: multi-scan (*SADABS*; Sheldrick, 2003 ▶) *T*_min_ = 0.639, *T*_max_ = 0.746
9074 measured reflections
2256 independent reflections
1973 reflections with *I* > 2σ(*I*)
*R*_int_ = 0.040

#### Refinement

*R*[*F* ^2^ > 2σ(*F* ^2^)] = 0.035
*wR*(*F* ^2^) = 0.086
*S* = 1.05
2256 reflections
151 parameters
2 restraints
H-atom parameters constrained
Δρ_max_ = 0.29 e Å^−3^
Δρ_min_ = −0.33 e Å^−3^

### 

Data collection: *COLLECT* (Hooft, 1998 ▶); cell refinement: *DENZO* (Otwinowski & Minor, 1997 ▶) and *COLLECT*; data reduction: *DENZO* and *COLLECT*; program(s) used to solve structure: *SHELXS97* (Sheldrick, 2008 ▶); program(s) used to refine structure: *SHELXL97* (Sheldrick, 2008 ▶); molecular graphics: *DIAMOND* (Brandenburg, 2006 ▶); software used to prepare material for publication: *publCIF* (Westrip, 2009 ▶).

## Supplementary Material

Crystal structure: contains datablocks global, I. DOI: 10.1107/S1600536809049654/lh2958sup1.cif

Structure factors: contains datablocks I. DOI: 10.1107/S1600536809049654/lh2958Isup2.hkl

Additional supplementary materials:  crystallographic information; 3D view; checkCIF report

## Figures and Tables

**Table 1:** Hydrogen-bond geometry (Å, °)

*D*—H⋯*A*	*D*—H	H⋯*A*	*D*⋯*A*	*D*—H⋯*A*
N3—H1n⋯N2^i^	0.88	2.25	3.0828 (19)	157
N3—H2n⋯N1^ii^	0.88	2.12	3.003 (2)	175
C3—H3a⋯N2^iii^	0.99	2.60	3.552 (2)	162
C3—H3b⋯S1^iv^	0.99	2.85	3.6687 (17)	141
C7—H7⋯O2^v^	0.95	2.53	3.355 (2)	145
C9—H9⋯O1^vi^	0.95	2.51	3.446 (2)	168
C5—H5⋯*Cg* ^iii^	0.95	2.86	3.7708 (17)	160

Symmetry codes: (i) 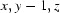; (ii) 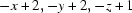; (iii) 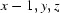; (iv) 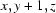; (v) 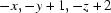; (vi) 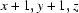. *Cg* is the centroid of the S1/N1/N2/C1/C2 ring.

**Fig. 1.**
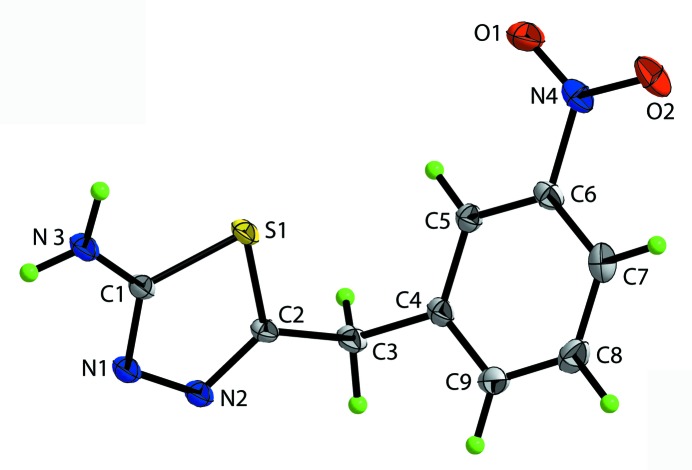
Molecular structure of (I) showing atom-labelling scheme and displacement ellipsoids at the 50% probability level.

**Fig. 2.**
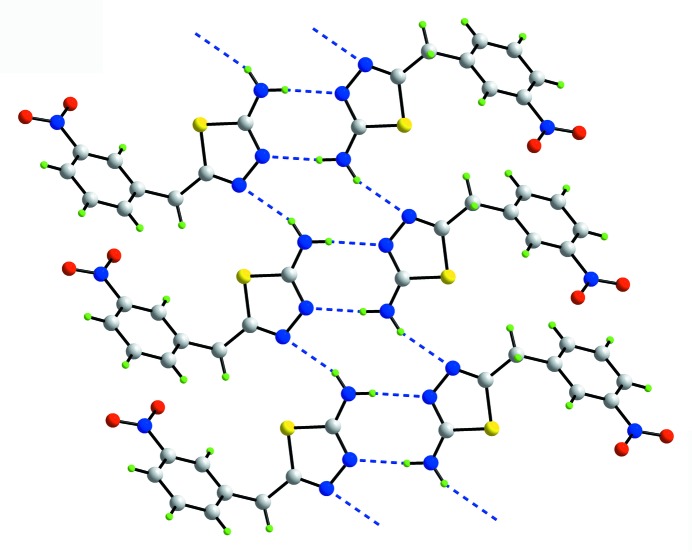
Supramolecular chain along [010] in (I) mediated by N–H···N hydrogen bonds (blue dashed lines). Colour code: S, yellow; O, red; N, blue; C, grey; and H, green.

**Fig. 3.**
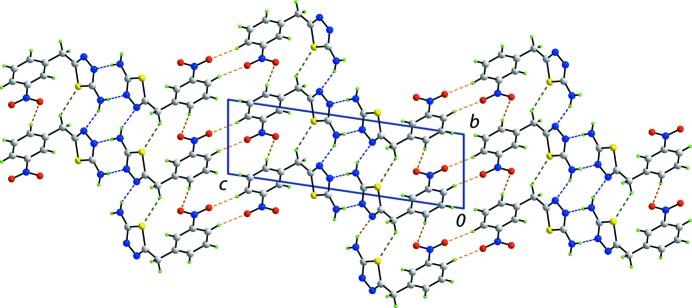
Unit-cell contents for (I) viewed in projection down the a axis. The N–H···N (blue), C—H···O (orange) and C—H···S (green) contacts are shown as dashed lines. Colour code: S, yellow; O, red; N, blue; C, grey; and H, green.

## supplementary crystallographic information

### Comment

1,3,4-Thiadiazoles have attracted much attention due to their biological activities (Thomasco *et al.*, 2003; Oruç *et al.*, 2004; Moise *et al.*, 2009; Amir *et al.*, 2009), with particular attention being paid to the anti-trypanosomal activities of Megazol, and related compounds (Carvalho *et al.*, 2004, 2008; Riente *et al.*, 2009: Poorrajab *et al.*, 2009). In continuation of our interests in 1,3,4-thiadiazoles (Boechat *et al.*, 2006, 2008; Carvalho *et al.*, 2004, 2008), we now report the structure of the title compound, (I), obtained by modification of a general procedure (Turner *et al.*, 1988).

In the molecular structure of (I) atom S1 is orientated towards the benzene ring, Fig. 1. The dihedral angle between the thiadiazole (r.m.s. deviation = 0.005 Å) and benzene (r.m.s. deviation = 0.004 Å) rings of 73.92 (8) ° indicates a twist between planes as seen in the S1–C2–C3–C4 torsion angle of 45.44 (18) °. The nitro group is effectively co-planar with the benzene ring to which it is attached as seen in the O1–N4–C6–C5 torsion angle of 6.3 (2) °.

The crystal packing is dominated by N—H···N hydrogen bonds. Each of the amine-H atoms connects to a centrosymmetrically related molecule leading to eight-membered {···HNCN}_2_ and 10-membered {···HNH···NN}_2_ synthons. Each synthon is planar and alternate in a supramolecular tape orientated along [010], Table 1 and Fig. 2. Chains are consolidated into a 3-D network by a combination of C—H···O, C—H···S and C—H···π interactions, Table 1 and Fig. 3.

### Experimental

A finely ground mixture of 2-nitrophenylacetic acid (0.49 g, 2.7 mmol) and thiosemicarbazide (0.25 g, 2.7 mmol) was added in portions over 0.5 h to polyphosphoric acid (5 g) at 353 K. The reaction mixture was maintained at 353 K for 5 h and cooled, water/ice was added, and the mixture was finally basified with NaOH 30% (aq.). The solids isolated by filtration were washed with water and air-dried to give (I), which was recrystallized from EtOH, m.p. 471–473 K; yield 72%. The sample used in the structure determination was obtained after a further recrystallization from EtOH. ^1^H NMR (d_6_-DMSO) δ: 4.47 (s, 2H, CH_2_), 7.05 (s, 2H, NH_2_), 7.55 (m, 2H, H4 and H5), 7.72 (m, 1H, H6), 8.03 (d, 1H, J = 8.0 Hz) p.p.m. ^13^C NMR (d_6_-DMSO) δ: 32.8, 124.7, 128.6, 132.0, 132.6, 133.8, 148.5, 155.1, 168.7 p.p.m.

### Refinement

The C-bound H atoms were geometrically placed with C—H = 0.95–0.99 Å, and refined as riding with *U*_iso_(H) = 1.2*U*_eq_(C). The N-bound H atoms were located from a difference map and included in the model with N–H = 0.880±0.001 Å, and with *U*_iso_(H) = 1.2*U*_eq_(N).

### Crystal data

**Table:**

C_9_H_8_N_4_O_2_S	*Z* = 2
*M**_r_* = 236.26	*F*(000) = 244
Triclinic, *P*1	*D*_x_ = 1.587 Mg m^−^^3^
Hall symbol: -P 1	Mo *K*α radiation, λ = 0.71073 Å
*a* = 5.0878 (2) Å	Cell parameters from 11753 reflections
*b* = 5.6213 (3) Å	θ = 2.9–27.5°
*c* = 17.8035 (9) Å	µ = 0.32 mm^−^^1^
α = 80.980 (3)°	*T* = 120 K
β = 85.677 (3)°	Block, colourless
γ = 79.855 (3)°	0.38 × 0.20 × 0.09 mm
*V* = 494.42 (4) Å^3^	

### Data collection

**Table:**

Nonius KappaCCD area-detector diffractometer	2256 independent reflections
Radiation source: Enraf Nonius FR591 rotating anode	1973 reflections with *I* > 2σ(*I*)
10 cm confocal mirrors	*R*_int_ = 0.040
Detector resolution: 9.091 pixels mm^-1^	θ_max_ = 27.5°, θ_min_ = 3.5°
φ and ω scans	*h* = −6→5
Absorption correction: multi-scan (*SADABS*; Sheldrick, 2003)	*k* = −7→7
*T*_min_ = 0.639, *T*_max_ = 0.746	*l* = −23→23
9074 measured reflections	

### Refinement

**Table:**

Refinement on *F*^2^	Primary atom site location: structure-invariant direct methods
Least-squares matrix: full	Secondary atom site location: difference Fourier map
*R*[*F*^2^ > 2σ(*F*^2^)] = 0.035	Hydrogen site location: inferred from neighbouring sites
*wR*(*F*^2^) = 0.086	H-atom parameters constrained
*S* = 1.05	*w* = 1/[σ^2^(*F*_o_^2^) + (0.0347*P*)^2^ + 0.3167*P*] where *P* = (*F*_o_^2^ + 2*F*_c_^2^)/3
2256 reflections	(Δ/σ)_max_ = 0.001
151 parameters	Δρ_max_ = 0.29 e Å^−^^3^
2 restraints	Δρ_min_ = −0.33 e Å^−^^3^

### Special details

**Table:**

Geometry. All s.u.'s (except the s.u. in the dihedral angle between two l.s. planes) are estimated using the full covariance matrix. The cell s.u.'s are taken into account individually in the estimation of s.u.'s in distances, angles and torsion angles; correlations between s.u.'s in cell parameters are only used when they are defined by crystal symmetry. An approximate (isotropic) treatment of cell s.u.'s is used for estimating s.u.'s involving l.s. planes.
Refinement. Refinement of *F*^2^ against ALL reflections. The weighted *R*-factor *wR* and goodness of fit *S* are based on *F*^2^, conventional *R*-factors *R* are based on *F*, with *F* set to zero for negative *F*^2^. The threshold expression of *F*^2^ > 2σ(*F*^2^) is used only for calculating *R*-factors(gt) *etc*. and is not relevant to the choice of reflections for refinement. *R*-factors based on *F*^2^ are statistically about twice as large as those based on *F*, and *R*- factors based on ALL data will be even larger.

### Fractional atomic coordinates and isotropic or equivalent isotropic displacement parameters (Å^2^)

**Table:**

	*x*	*y*	*z*	*U*_iso_*/*U*_eq_	
S1	0.39352 (8)	0.87897 (7)	0.63466 (2)	0.01803 (12)	
O1	−0.3420 (3)	0.5966 (2)	0.79836 (7)	0.0282 (3)	
O2	−0.2766 (3)	0.4981 (2)	0.91883 (7)	0.0326 (3)	
N1	0.7365 (3)	1.1393 (2)	0.56903 (8)	0.0187 (3)	
N2	0.5591 (3)	1.2890 (2)	0.61221 (8)	0.0185 (3)	
N3	0.8153 (3)	0.7356 (2)	0.54139 (8)	0.0198 (3)	
H1N	0.7552	0.5959	0.5485	0.030*	
H2N	0.9445	0.7652	0.5073	0.030*	
N4	−0.2396 (3)	0.6204 (2)	0.85639 (8)	0.0218 (3)	
C1	0.6754 (3)	0.9177 (3)	0.57569 (9)	0.0160 (3)	
C2	0.3731 (3)	1.1810 (3)	0.64921 (9)	0.0161 (3)	
C3	0.1583 (3)	1.2978 (3)	0.70123 (9)	0.0189 (3)	
H3A	−0.0131	1.3369	0.6754	0.023*	
H3B	0.2049	1.4528	0.7116	0.023*	
C4	0.1235 (3)	1.1329 (3)	0.77600 (9)	0.0167 (3)	
C5	−0.0430 (3)	0.9582 (3)	0.78225 (9)	0.0164 (3)	
H5	−0.1411	0.9442	0.7403	0.020*	
C6	−0.0634 (3)	0.8046 (3)	0.85083 (9)	0.0180 (3)	
C7	0.0726 (3)	0.8188 (3)	0.91408 (10)	0.0231 (4)	
H7	0.0545	0.7117	0.9604	0.028*	
C8	0.2360 (3)	0.9947 (3)	0.90746 (10)	0.0249 (4)	
H8	0.3309	1.0096	0.9500	0.030*	
C9	0.2626 (3)	1.1498 (3)	0.83935 (10)	0.0208 (3)	
H9	0.3766	1.2684	0.8358	0.025*	

### Atomic displacement parameters (Å^2^)

**Table:**

	*U*^11^	*U*^22^	*U*^33^	*U*^12^	*U*^13^	*U*^23^
S1	0.0196 (2)	0.0135 (2)	0.0218 (2)	−0.00748 (15)	0.00547 (15)	−0.00271 (14)
O1	0.0321 (7)	0.0250 (6)	0.0306 (7)	−0.0141 (5)	−0.0028 (5)	−0.0022 (5)
O2	0.0328 (7)	0.0306 (7)	0.0307 (7)	−0.0112 (6)	0.0028 (6)	0.0113 (5)
N1	0.0184 (7)	0.0156 (6)	0.0228 (7)	−0.0061 (5)	0.0044 (5)	−0.0039 (5)
N2	0.0186 (7)	0.0152 (6)	0.0225 (7)	−0.0061 (5)	0.0034 (5)	−0.0036 (5)
N3	0.0206 (7)	0.0140 (6)	0.0252 (8)	−0.0067 (5)	0.0064 (5)	−0.0036 (5)
N4	0.0193 (7)	0.0170 (7)	0.0271 (8)	−0.0029 (5)	0.0029 (6)	0.0012 (6)
C1	0.0156 (7)	0.0165 (7)	0.0162 (8)	−0.0058 (6)	−0.0008 (6)	0.0001 (6)
C2	0.0188 (7)	0.0130 (7)	0.0174 (8)	−0.0060 (6)	−0.0006 (6)	−0.0014 (6)
C3	0.0214 (8)	0.0131 (7)	0.0225 (8)	−0.0052 (6)	0.0035 (6)	−0.0024 (6)
C4	0.0143 (7)	0.0145 (7)	0.0210 (8)	−0.0018 (6)	0.0046 (6)	−0.0049 (6)
C5	0.0156 (7)	0.0163 (7)	0.0170 (8)	−0.0020 (6)	0.0006 (6)	−0.0033 (6)
C6	0.0150 (7)	0.0156 (7)	0.0226 (8)	−0.0024 (6)	0.0025 (6)	−0.0020 (6)
C7	0.0226 (8)	0.0262 (9)	0.0174 (8)	0.0002 (7)	0.0019 (6)	−0.0001 (7)
C8	0.0228 (9)	0.0325 (9)	0.0209 (9)	−0.0032 (7)	−0.0041 (7)	−0.0085 (7)
C9	0.0173 (8)	0.0220 (8)	0.0254 (9)	−0.0052 (6)	0.0017 (6)	−0.0096 (7)

### Geometric parameters (Å, °)

**Table:**

S1—C1	1.7373 (16)	C3—H3A	0.9900
S1—C2	1.7412 (15)	C3—H3B	0.9900
O1—N4	1.2271 (19)	C4—C5	1.393 (2)
O2—N4	1.2321 (18)	C4—C9	1.400 (2)
N1—C1	1.321 (2)	C5—C6	1.389 (2)
N1—N2	1.3949 (19)	C5—H5	0.9500
N2—C2	1.297 (2)	C6—C7	1.385 (2)
N3—C1	1.342 (2)	C7—C8	1.386 (2)
N3—H1N	0.8800	C7—H7	0.9500
N3—H2N	0.8799	C8—C9	1.391 (2)
N4—C6	1.472 (2)	C8—H8	0.9500
C2—C3	1.506 (2)	C9—H9	0.9500
C3—C4	1.516 (2)		
			
C1—S1—C2	87.36 (7)	H3A—C3—H3B	107.9
C1—N1—N2	111.82 (13)	C5—C4—C9	118.89 (15)
C2—N2—N1	113.45 (12)	C5—C4—C3	120.45 (14)
C1—N3—H1N	117.3	C9—C4—C3	120.64 (14)
C1—N3—H2N	119.9	C6—C5—C4	118.95 (14)
H1N—N3—H2N	122.0	C6—C5—H5	120.5
O1—N4—O2	123.37 (14)	C4—C5—H5	120.5
O1—N4—C6	118.11 (13)	C7—C6—C5	122.94 (15)
O2—N4—C6	118.52 (14)	C7—C6—N4	118.81 (14)
N1—C1—N3	124.39 (14)	C5—C6—N4	118.25 (14)
N1—C1—S1	113.68 (12)	C6—C7—C8	117.65 (15)
N3—C1—S1	121.93 (11)	C6—C7—H7	121.2
N2—C2—C3	124.72 (13)	C8—C7—H7	121.2
N2—C2—S1	113.68 (12)	C9—C8—C7	120.82 (15)
C3—C2—S1	121.59 (11)	C9—C8—H8	119.6
C2—C3—C4	112.03 (13)	C7—C8—H8	119.6
C2—C3—H3A	109.2	C8—C9—C4	120.74 (15)
C4—C3—H3A	109.2	C8—C9—H9	119.6
C2—C3—H3B	109.2	C4—C9—H9	119.6
C4—C3—H3B	109.2		
			
C1—N1—N2—C2	−0.19 (19)	C3—C4—C5—C6	177.85 (14)
N2—N1—C1—N3	−178.79 (14)	C4—C5—C6—C7	0.8 (2)
N2—N1—C1—S1	0.65 (17)	C4—C5—C6—N4	−179.81 (13)
C2—S1—C1—N1	−0.70 (12)	O1—N4—C6—C7	−174.26 (14)
C2—S1—C1—N3	178.76 (14)	O2—N4—C6—C7	5.7 (2)
N1—N2—C2—C3	178.85 (14)	O1—N4—C6—C5	6.3 (2)
N1—N2—C2—S1	−0.36 (17)	O2—N4—C6—C5	−173.78 (14)
C1—S1—C2—N2	0.59 (12)	C5—C6—C7—C8	−0.2 (2)
C1—S1—C2—C3	−178.64 (14)	N4—C6—C7—C8	−179.63 (15)
N2—C2—C3—C4	−133.70 (16)	C6—C7—C8—C9	−0.4 (3)
S1—C2—C3—C4	45.44 (18)	C7—C8—C9—C4	0.5 (3)
C2—C3—C4—C5	−85.79 (18)	C5—C4—C9—C8	0.1 (2)
C2—C3—C4—C9	92.73 (17)	C3—C4—C9—C8	−178.44 (15)
C9—C4—C5—C6	−0.7 (2)		

### Hydrogen-bond geometry (Å, °)

**Table:**

*D*—H···*A*	*D*—H	H···*A*	*D*···*A*	*D*—H···*A*
N3—H1n···N2^i^	0.88	2.25	3.0828 (19)	157
N3—H2n···N1^ii^	0.88	2.12	3.003 (2)	175
C3—H3a···N2^iii^	0.99	2.60	3.552 (2)	162
C3—H3b···S1^iv^	0.99	2.85	3.6687 (17)	141
C7—H7···O2^v^	0.95	2.53	3.355 (2)	145
C9—H9···O1^vi^	0.95	2.51	3.446 (2)	168
C5—H5···Cg^iii^	0.95	2.86	3.7708 (17)	160

Symmetry codes: (i) *x*, *y*−1, *z*; (ii) −*x*+2, −*y*+2, −*z*+1; (iii) *x*−1, *y*, *z*; (iv) *x*, *y*+1, *z*; (v) −*x*, −*y*+1, −*z*+2; (vi) *x*+1, *y*+1, *z*.
